# Report on the Importance and Economy of Sanitary Measures to Cities

**Published:** 1861-09

**Authors:** 


ART. XI.—*Report on the Importance and Economy of Sanitary Measures to Cities.* By JOHN BELL, M.D., of Philadelphia. (Board of Councilmen, Nov. 28, 1859.) Doc. No. 20, New York, 1860.

*Draft of a Sanitary Code for Cities, (Reported to the Committee on Internal Hygiene.)* By HENRY G. CLARK, M.D., of Boston, one of the Committee.

*Report upon Sewerage, Water Supply, and Offal.* By JOHN M. GRISCOM, M.D., of New York.

THE three great cities of the North and East are here found in a very sisterly manner inquiring after each other's health. Philadelphia shows how necessary it is to the sanitary condition of her own people and those of every other city to attend to ventilation, sewerage, and offensive emanations of all kinds. Boston coincides with her hygienic views, and furnishes the plan which she thinks best adapted for preserving the public health, so that no fastidious nose shall be offended, and nothing but pleasure to the senses flow into every channel of communication with the external world. New York very modestly comes in at the end of the book with her views upon special subjects of sewerage, water supply, etc.; minor, but very important subdivisions under the general head of sanitary regulations, which form the grand subject of the whole volume before us.

The report of Dr. Bell is rather too lengthy for popular reading, although it is full of valuable information as to the progress of sanitary measures from the earliest times. We should have been glad to see at the end of this report a summary of the interesting facts with which it abounds, from which we might have quoted some of the author's conclusions, but in its absence we must content ourselves with a brief notice of its objects and its facts.

Beginning with ancient Egyptian, Carthagenian, and Roman hygiene, he speaks with confidence of the amelioration of the sanitary condition of cities which accompanied advancing civilization.

"The mortality in Paris in the early part of the fourteenth century has been estimated, from a manuscript document, to be 1 in 20; whereas the average mortality of the inhabitants of that city, in the very poorest arrondissement or ward, as we might term it, in which poverty and destitution are extreme, was 1 in 24, in the first third of the present century. The average deaths in all Paris at the same date (1830) was 1 in 32, and among the more wealthy inhabitants, 1 in 42. * * * M. Marc d'Espine, in a late work on 'Comparative Mortuary Statistics,' shows that the *probable* life at Geneva in the sixteenth century was rather less than 5 years; in the seventeenth century, 11 years; at the beginning of the eighteenth century, 27 years; at the end of the century, 32 years; and now it is 44 years. M. Mallet had previously ascertained, from the same documents, that the mean duration of life in Geneva, in 1833, was nearly the double (or as 40 years 5 months to 21 years 2 months) of that recorded rather more than two centuries before."

At this rate of progress, we should scarcely be surprised if statisticians would try to convince us that in the course of the next few centuries we may look upon octogenarians as young men with a long future before them.

The usual arguments in favor of a complete system of sewerage are detailed; but the points which principally interest us in this connection, such as the effect of the sewerage gases on the animal system, are quoted from the able report of Dr. Letheby of London. Cases of chronic poisoning produced by the inhalation of very small quantities of sewer miasm are given, as well as the means of remedying such miasms or of preventing the offensive and toxical effects of the emanations from sewers by disinfectants and deodorizers.

Dr. Bell then discusses at length the difficult subject of ventilation, with reference especially to the cellar and alley life of our large cities. The general want of ventilation in schools, hospitals, printing-offices, and factories is referred to, the defect of light, and the remedies for these conditions. The different modes of ventilation usually adopted and the merits of each are discussed. All artificial ventilation is accomplished in one of three ways: 1st, by heat or by a chemical process; 2d, by pumping, or a mechanical process; or 3d, by the pressure and movements of the atmosphere, without let or hinderance. We think with the author that "the great number of plans for ventilation would imply that an easy and efficient system is not yet reached."

Water supply, intemperance, sanitary improvements, pulmonary and cutaneous purification, nuisances, and interments in cities, form the subject-matter of the balance of the book, but are treated at such length that we can merely refer to them without quoting any passages. The whole report teems with interesting general information, and we know of no one who, having a subject given to him with such wide scope for voluminousness, could more ably than Dr. Bell bring together an immense mass of materials from all available sources.

The Reports of Drs. Clark and Griscom are also to be commended as efforts directed in like manner to the preservation of the public health.

